# Inverse numerical modeling for predicting kinetic rate constants in polyolefin pyrolysis based on product yield distribution for efficient plastic recycling

**DOI:** 10.1038/s41598-025-31977-0

**Published:** 2025-12-11

**Authors:** Rao Adeel Un Nabi, Rashid Ul Haq, Muhammad Adnan, TieJun Wang, Hassan Abbas Khawaja

**Affiliations:** 1 State Key Laboratory of Ultra Intense Laser Science and Technology, Shanghai Institute of Optics and Fine Mechanics, Chinese Academy of Sciences, Shanghai, 201800 China; 2 Center of Materials Science and Optoelectronics Engineering, University of Chinese Academy of Sciences, Beijing, 100049 China; 3 National Laboratory on High Power Laser and Physics, Shanghai Institute of Optics and Fine Mechanics, Chinese Academy of Sciences, Shanghai, 201800 China; 4 State Key Laboratory of Infrared Physics, Shanghai Institute of Technical Physics, Chinese Academy of Sciences, Shanghai, 200083 China; 5 Department of Automation and Process Engineering, Faculty of Engineering Science and Technology, UiT The Arctic University of Norway, 9019 Tromsø, Norway

**Keywords:** HDPE, Kinetic rate constants, Pyrolysis, Inverse numerical modeling, Pyrolytic oil, Chemistry, Energy science and technology, Engineering, Environmental sciences, Materials science, Mathematics and computing

## Abstract

The prediction of kinetic rate constants is a significant challenge in effective plastic pyrolysis recycling due to the wide range of generated byproducts, including char and aromatics. To address this challenge, an inverse numerical modeling methodology aimed at selective product distribution was formulated and assessed utilizing an established polyolefins reaction mechanism from existing literature to attain target yields of 50% oil and 50% gas within 60 min. This was accomplished by minimizing byproduct formation through an objective function designed to reduce the squared divergence between simulated and desired outputs, which was solved using an ordinary differential equation solver (ode23). The technique predicts rate constants to attain the desired fit, which was verified by experimental yields at 450 and 500 °C. Results indicate that inverse numerical modeling for precise estimation of kinetic rate constants markedly enhances the correlation between predicted and experimental yields, corresponding to a consistent yield trend by minimizing wax production at a reasonable scale throughout the processing time. The predicted rate constants suggest a 5% higher gas yield by eliminating wax up to 5% in line with maintaining the oil yield efficiency. Literature data support these findings and offer recommendations for enhancing experimental and statistical methodologies for predicting kinetic rate constants. This methodology provides a solid foundation for precisely predicting and optimizing product distributions in plastic pyrolysis across various temperature conditions, which could serve as an alternative and practical approach for predicting rate constants.

## Literature overview

Although extensive qualitative and quantitative data are available regarding pyrolysis reaction mechanisms, kinetic models, and associated rate constants, this information has not been integrated adequately to enhance the process. The literature indicates a notable lack of predictive, yield-targeted approaches to determining the kinetic rate constants, which consequently affect product yields directly^1^. Inverse kinetic modeling, a numerical process that determines kinetic rate constants based on specific product outcomes, remains largely unstudied. This is mainly attributable to the insufficiency of both experimentally derived and statistically estimated constants in achieving satisfactory product yields, suggesting a need to reevaluate the methodologies for establishing these rate constants^2^.

Several experimental, statistical, and numerical methodologies have been established to optimize kinetic rate constants^3^. Despite these models improving oil yields and refining kinetic precision, however, still demonstrate considerable limits, particularly in managing end product distributions and adapting to variable conditions^4^. Numerous approaches rely significantly on empirical data, lacking a proactive framework that facilitates real-time adjustments to rate constants in alignment with yield objectives. This constraint underscores the need for an inverse numerical modeling method that determines kinetic rate constants in accordance with specified yield targets^5^. This methodology may enhance the dependability, predictive capacity, and economic feasibility of HDPE pyrolysis optimization, particularly by focusing on objectives such as maximizing oil recovery or minimizing gas production^6^. Advancements in numerical modeling enable the strategic application of established reaction mechanism data to predict kinetic rate constants that align with targeted product distributions, such as an optimal oil-to-gas ratio, rather than relying on predetermined values^7^.

According to the literature cited over the last 10 years, experimental studies indicate that Salem et al.^8^. investigated the thermal degradation of HDPE using isothermal pyrolysis in a microthermal-balance reactor, deriving kinetic rate constants and the overall activation energy (E₀). In another investigation, Salem et al.^9^. employed a fixed-bed batch reactor to examine gasoline-range hydrocarbons at temperatures ranging from 500 to 800 °C under nitrogen, achieving a peak oil yield of 70% at 550 °C. Zeaiter et al.^10^. examined the thermo-catalytic cracking of polyethylene bottles to evaluate the feasibility of continuous operation, identifying products such as paraffins, olefins, and aromatics. Comparably, Alqarni et al.^11^. utilized R-software to analyze previous research data and predict kinetic rate constants by determining optimal combinations of activation energy and frequency factor within a temperature range of 370 °C to 410 °C, resulting in an impressive 99% oil yield. Nabi et al.^12^. conducted a sensitivity analysis on statistically predicted rate constants, identifying k7 as the most effective of the nine evaluated constants. Irfan et al.^13^. employed a Box–Behnken design and response surface methodology (RSM) with Design-Expert software to predict rate constants at 500 °C and 550 °C statistically. Their projected constants yielded an 85% oil output and less than 1% byproducts, exceeding the 32% oil yield from conventional experimental constants. This suggests that the expected rate constants at 550 °C are advantageous for the industrial-scale production of liquid fuels from HDPE. Moreover, Irfan et al.^14^. identified through additional sensitivity analysis that k_8_ could serve as a pivotal kinetic constant for efficient recycling.

The aforementioned literature and findings suggest that existing experimental and statistical techniques for determining kinetic rate constants are often inadequate in yield optimization. A methodological transformation is essential, involving the systematic utilization of established chemical pathways for plastic pyrolysis. Integrating inverse numerical modeling based on yield distribution into these pathways could facilitate more accurate predictions of rate constants, hence enhancing the regulation of product distributions and reducing the difference between theoretical and real implementations. This process could provide considerable potential to bridge the gap between predicted and actual product yields in plastic pyrolysis. These findings contradict previous optimization and sensitivity studies (11–14) that employed algebraic or regression techniques such as RSM and statistical calibration. The current study proposes a yield-based inverse modeling framework. In this methodology, the kinetic rate constants are not obtained through regression of experimental data but are iteratively adjusted to achieve pre-specified yield targets by minimizing the least-squares difference between simulated and desired outputs. This formulation establishes a direct relationship between the observed product distribution and the kinetic parameters, providing a more flexible and predictive approach than conventional statistical or Bayesian calibration methods.

This study will apply the established polyolefins pyrolysis reaction mechanism, as described in the literature, at temperatures of 450 °C and 500 °C using inverse kinetic modeling to determine the corresponding kinetic rate constants. A lumped kinetic model will be utilized, consisting of four pseudo-components: polyolefins (HDPE, LDPE, and PP), wax, gas, and oil. The model will incorporate six first-order processes that illustrate the thermal deterioration of HDPE and the ensuing cracking of intermediate species, such as waxes. The initial kinetic rate constants will serve as input parameters, with the modeling framework aiming for a product yield of 50% oil and 50% gas over a simulated reaction period of 60 min. An objective function will be formulated to minimize the squared divergence between simulated and target yields, and the system will be solved using ordinary differential equations (ode23). The accuracy and reliability of the computed kinetic rate constants will be evaluated against actual data obtained during the reaction period to determine their effectiveness in controlling final product distribution. This approach could provide flexibility for incorporating additional product streams or complex reaction networks, serving as an alternative to empirically or theoretically derived rate constants to enhance the performance of plastic pyrolysis processes.

### Highlights

1. Yield-based estimation of kinetic rate constants via inverse numerical modeling.
2. Objective function applied to minimize errors between simulated and target yields.
3. Comparison of actual and predicted rate constants from product yield results.

## Model framework

**Fig. 1:**
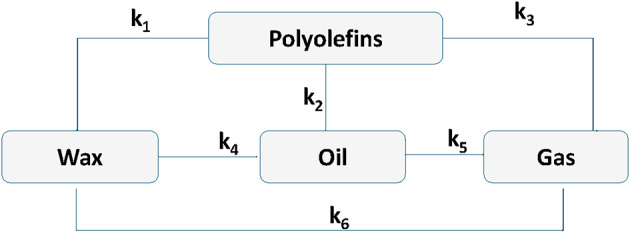
The polyolefins pyrolysis reaction pathways.

The reaction mechanism and experimental or actual rate constants were considered from this literature for investigation in this study^15^. The reaction rate constants used in this study were taken from previous polyolefin pyrolysis experiments conducted under a nitrogen atmosphere using batch fixed-bed reactors, while neglecting the effects of mass-transfer limitations. The current inverse model was employed to replicate these batch-type kinetic conditions under the ideal assumption of a homogeneous reaction. Inverse modeling was performed using the ODE23s solver in MATLAB, with the initial experimental rate constants as input values. The optimized or predicted constants were obtained by minimizing the sum of squares between the simulated and reference product yields using a least-squares algorithm. This approach ensures consistency between the kinetic environment of the experiment and the simulation framework. According to this study, the pyrolysis of polyolefins (HDPE, LDPE, PP) is described in a lumped kinetic model, which classifies the products into four categories: polymer (P), wax (W), liquid oil (L), and gas (G). The conversion is dictated by six primary and secondary pathways (k₁–k₆), for which the parameter values are documented at 450 °C and 500 °C (Eqs. 1–4). These values served as baseline inputs and initial conditions for parameter optimization. An objective function was applied to the refined kinetic parameters to achieve the estimated targeted values for the yields of oil (approximately 50%) and gas (approximately 45%). The governing equations were numerically solved in MATLAB using the ODE solver ode23s, and results were compared between original and optimized rate constants. This reaction network is depicted in the reaction scheme (Fig. 1), which shows that polyolefins decompose into wax (k₁), oil (k₂), or gas (k₃). Meanwhile, secondary conversions of wax (k₄) or oil (k₆) to gas (k₅) also occur, leading to oil gas.

1 $$\:\frac{dP}{dt}=\:-\left({k}_{1}+{k}_{2}+{k}_{3}\right)P$$

2 $$\:\frac{dW}{dt}=\:{k}_{1\:}\mathrm{P}-\left({k}_{4}+{k}_{6}\right)W$$

3 $$\:\frac{dL}{dt}=\:{k}_{2\:}\mathrm{P}+{k}_{4}W-{k}_{5}L$$

4 $$\:\frac{dG}{dt}=\:{k}_{3}\mathrm{P}+{\mathrm{k}}_{5}L+{k}_{6}W$$

**Fig. 2:**
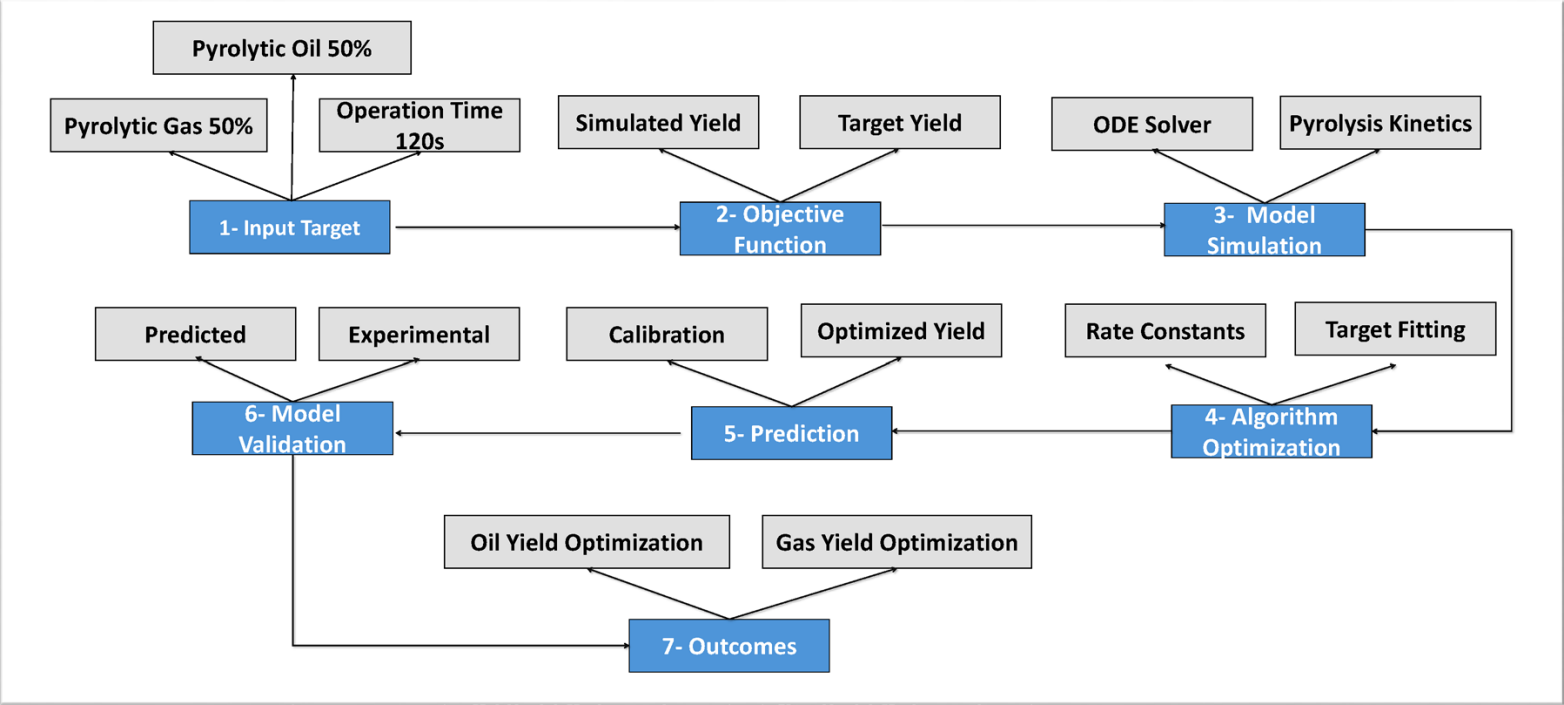
The inverse numerical model framework for the prediction of rate constants.

The model architecture used to determine the kinetic rate constants is illustrated in Fig. 2. The principal modeling sequence (Steps 1–7) is presented in blue, while the supporting elements such as input data, simulated yields, and calibration steps are shown in grey. This visual distinction separates the main logical workflow from supplementary information, thereby improving clarity and overall readability.The product distribution, such as 50% oil and 50% gas yield targets, was achieved during model calibration within a duration of 60 min. The time-dependent evolution of species during HDPE pyrolysis utilizes the stiff ODE solver ode23s. This solver resolves the complexities associated with highly coupled and stiff nonlinear systems, such as thermal degradation of HDPE. The defined objective was to minimize the difference between model results and target values of oil and gas yields, as described in Eqs. 5 and 6, where L(t_end_;k) and G(t_end_;k) indicate simulated liquid oil and gas yields, and L_target_=50%, G_target_=50% represent the experimental conditions. The numerical inversion was conducted using a single-objective least-squares regression to reduce the discrepancy between the simulated and actual oil/gas yields. The objective functions are formulated in Eqs. (7) and (8), respectively. Here, L_sim_​ and G_sim_​ represent the simulated oil and gas yields at the end of the reaction time t_end_, while L_target_ and G_target_​ are the target yield values fixed at 50% oil and 50% gas, respectively. The optimized kinetic parameters were estimated by minimizing the total error function over the vector of rate constants k using the least-squares approach. This single-objective problem was solved iteratively for all polymer types using the ODE23s solver in MATLAB until convergence was achieved. The Eqs. 9–11 were employed to forecast the capability of the inverse numerical model was assessed quantitatively using the coefficient of determination (R²), root mean square error (RMSE), and mean absolute deviation (MAD). Y_exp_ and Y_pred_ represent the experimental and predicted yields, respectively, and n is the total data points. These statistical measures were derived from the expected and experimental oil–gas yield distributions at both temperature conditions. Table 1 presents the calculated values of these metrics, showing excellent agreement between the predicted and experimental data. This validates that the inverse model effectively replicates the thermal decomposition trends of polyolefins.

5 $${\mathrm{r}}\left( {\mathrm{k}} \right) = \left[ {\begin{array}{*{20}c} {{\mathrm{L}}\left( {{\mathrm{t}}_{{{\mathrm{end}}}} ;{\mathrm{k}}} \right){\mathrm{~~~~}} - } & {{\mathrm{L}}_{{{\mathrm{target}}}} } \\ {{\mathrm{G}}\left( {{\mathrm{t}}_{{{\mathrm{end}}}} ;{\mathrm{k}}} \right)~~~~~ - } & {G_{{target}} } \\ \end{array} } \right]$$

6 $${\mathrm{j}}\left( {\mathrm{k}} \right) = \left\| {r\left( k \right)} \right\|\begin{array}{*{20}c} 2 \\ 2 \\ \end{array}$$

7 $$\:r\left(k\right)=\left[{L}_{sim}\right({t}_{end},k)-{L}_{target}]$$

8 $$\:j\left(k\right)=\left[{G}_{sim}\right(tend,k)-{G}_{target}]$$

9 $$\:{R}^{2}=1-\frac{{\sum\:i({Y}_{exp}\:\:,i-{Y}_{exp})}^{2}}{{\sum\:i({Y}_{exp},i-{Y}_{pred},i)}^{2}}$$

10 $$\:RMSE=\sqrt{\frac{1}{n}\:{\sum\:{}_{i}\left({Y}_{exp,\:i}-{Y}_{pred,i}\right)}^{2\:}}$$

11 $$\:MAD=\frac{1}{n}{\sum\:}_{i}\mid\:{Y}_{exp,i}-{Y}_{pred,\:i}\mid\:$$

**Table 1:** The calculated R^2^, RMSE, and MAD values estimate the forecasting capability.

Polymer	Temperature (°C)	*R*²	RMSE (%)	MAD (%)
HDPE	450	0.987	2.45	1.92
	500	0.991	1.87	1.35
LDPE	450	0.982	2.93	2.41
	500	0.989	2.11	1.68
PP	450	0.985	2.76	2.04
	500	0.992	1.74	1.32

The optimization was performed using a gradient-based least-squares algorithm in MATLAB. The termination criterion was defined as a relative change in the objective function of less than 1 × 10^− 6^ over consecutive iterations. The rate constants were constrained to positive values, representing the physically reasonable condition. Baseline initial parameter values were obtained from literature-reported experimental rate constants^15^ for each polymer and temperature. The parameters were updated iteratively until convergence, ensuring that the resulting constants were numerically stable and reproducible. Additionally, mass balance closure was confirmed for all lumped species (polymer, wax, oil, and gas) at each simulation time interval. The sum of the yield fractions was held constant at 1.00 during the reaction for all components and the overall activity. This indicates that no material was lost during conversion, thereby verifying the overall mass conservation of the model.

Similar to other optimization problems, the objective function is minimized in an m-dimensional space using a stepwise process that requires extensive iterative evaluations, resulting in a substantial number of repetitions. The function typically yields inadequate predictions from the initial values. The objective function, defined as the discrepancy between the observed and target yields, is calculated. Subsequently, improvements are made through iterative parameter optimization rules. On average, between 6 and 22 iterations are needed for convergence, which usually depends on the nature of the data sets. Each iteration yields a near-maximum or near-minimum value contingent upon the specified step size and the constraints imposed by the function. The optimization process and the interface with the ODE solver ensured numerical consistency for each iteration. All executed trials at each temperature exhibited convergence, validating the reliability and reproducibility of the procedure. The optimized values for each operating state demonstrated temperature dependence, indicating that the fitted constants accurately represent the intrinsic kinetics of pyrolysis. The actual and predicted rate constant values are reported below in Table 2, and the detailed outcomes comparison can be found in the following section. The optimized iterative evaluations and convergence status obtained from inverse numerical modeling are evident in the pseudo-code reported in Table 3.

**Table 2:** Initial literature-reported rate constants and inverse-model-predicted kinetic rate constants as a function of temperature and plastic type.

Polymer	Temp (°C)	k₁	k₂	k₃	k₄	k₅	k₆
HDPE	450	1.70 × 10⁻¹	1.55 × 10⁻¹	2.44 × 10⁻⁹	1.35 × 10⁻¹	4.00 × 10⁻³	2.10 × 10⁻²
HDPE	500	5.45 × 10⁻¹	5.11 × 10⁻¹	2.43 × 10⁻⁸	4.29 × 10⁻¹	1.60 × 10⁻²	8.10 × 10⁻²
LDPE	450	1.46 × 10⁻¹	2.64 × 10⁻⁷	3.12 × 10⁻⁵	1.08 × 10⁻¹	9.00 × 10⁻³	4.00 × 10⁻³
LDPE	500	4.79 × 10⁻¹	1.95 × 10⁻⁶	1.72 × 10⁻⁴	3.75 × 10⁻¹	3.60 × 10⁻²	1.70 × 10⁻²
PP	450	1.88 × 10⁻¹	1.43 × 10⁻¹	3.30 × 10⁻²	2.60 × 10⁻¹	4.00 × 10⁻³	2.00 × 10⁻¹⁰
PP	500	6.06 × 10⁻¹	4.37 × 10⁻¹	1.17 × 10⁻¹	8.53 × 10⁻¹	1.60 × 10⁻²	2.33 × 10⁻⁹

**Table 3:** The optimized iterative evaluations and convergence status obtained from inverse numerical modeling.

Polymer	Rate Constant	450 °C (Initial)	450 °C (Fitted)	500 °C (Initial)	500 °C (Fitted)	Convergence status
HDPE	k₁	1.70 × 10⁻¹	2.94 × 10⁻¹	5.45 × 10⁻¹	3.42 × 10⁻¹	Converged
	k₂	1.55 × 10⁻¹	9.31 × 10⁻²	5.11 × 10⁻¹	1.29 × 10⁰	Converged
	k₃	2.44 × 10⁻⁹	3.13 × 10⁻⁹	2.43 × 10⁻⁸	1.93 × 10⁻⁷	Converged
	k₄	1.35 × 10⁻¹	2.61 × 10⁻²	4.29 × 10⁻¹	4.74 × 10⁻¹	Converged
	k₅	4.00 × 10⁻³	5.00 × 10⁻³	1.60 × 10⁻²	1.00 × 10⁻²	Converged
	k₆	2.10 × 10⁻²	2.18 × 10⁻²	8.10 × 10⁻²	5.80 × 10⁻²	Converged
LDPE	k₁	1.46 × 10⁻¹	1.09 × 10⁻¹	4.79 × 10⁻¹	1.25 × 10⁻¹	Converged
	k₂	2.64 × 10⁻⁷	1.05 × 10⁻⁷	1.95 × 10⁻⁶	1.92 × 10⁻⁷	Converged
	k₃	3.12 × 10⁻⁵	3.21 × 10⁻⁵	1.72 × 10⁻⁴	5.24 × 10⁻⁴	Converged
	k₄	1.08 × 10⁻¹	6.00 × 10⁻²	3.75 × 10⁻¹	5.50 × 10⁻²	Converged
	k₅	9.00 × 10⁻³	1.50 × 10⁻²	3.60 × 10⁻²	1.40 × 10⁻²	Converged
	k₆	4.00 × 10⁻³	5.00 × 10⁻³	1.70 × 10⁻²	6.00 × 10⁻³	Converged
PP	k₁	1.88 × 10⁻¹	1.10 × 10⁻¹	6.06 × 10⁻¹	9.54 × 10⁻¹	Converged
	k₂	1.43 × 10⁻¹	7.80 × 10⁻²	4.37 × 10⁻¹	8.60 × 10⁻¹	Converged
	k₃	3.30 × 10⁻²	5.80 × 10⁻²	1.17 × 10⁻¹	7.20 × 10⁻²	Converged
	k₄	2.60 × 10⁻¹	4.93 × 10⁻¹	8.53 × 10⁻¹	5.47 × 10⁻¹	Converged
	k₅	4.00 × 10⁻³	7.00 × 10⁻³	1.60 × 10⁻²	1.00 × 10⁻²	Converged
	k₆	2.00 × 10⁻¹⁰	5.80 × 10⁻¹²	2.33 × 10⁻⁹	2.03 × 10⁻⁷	Converged

## Results and discussion

### Model evaluation and validation

**Fig. 3:**
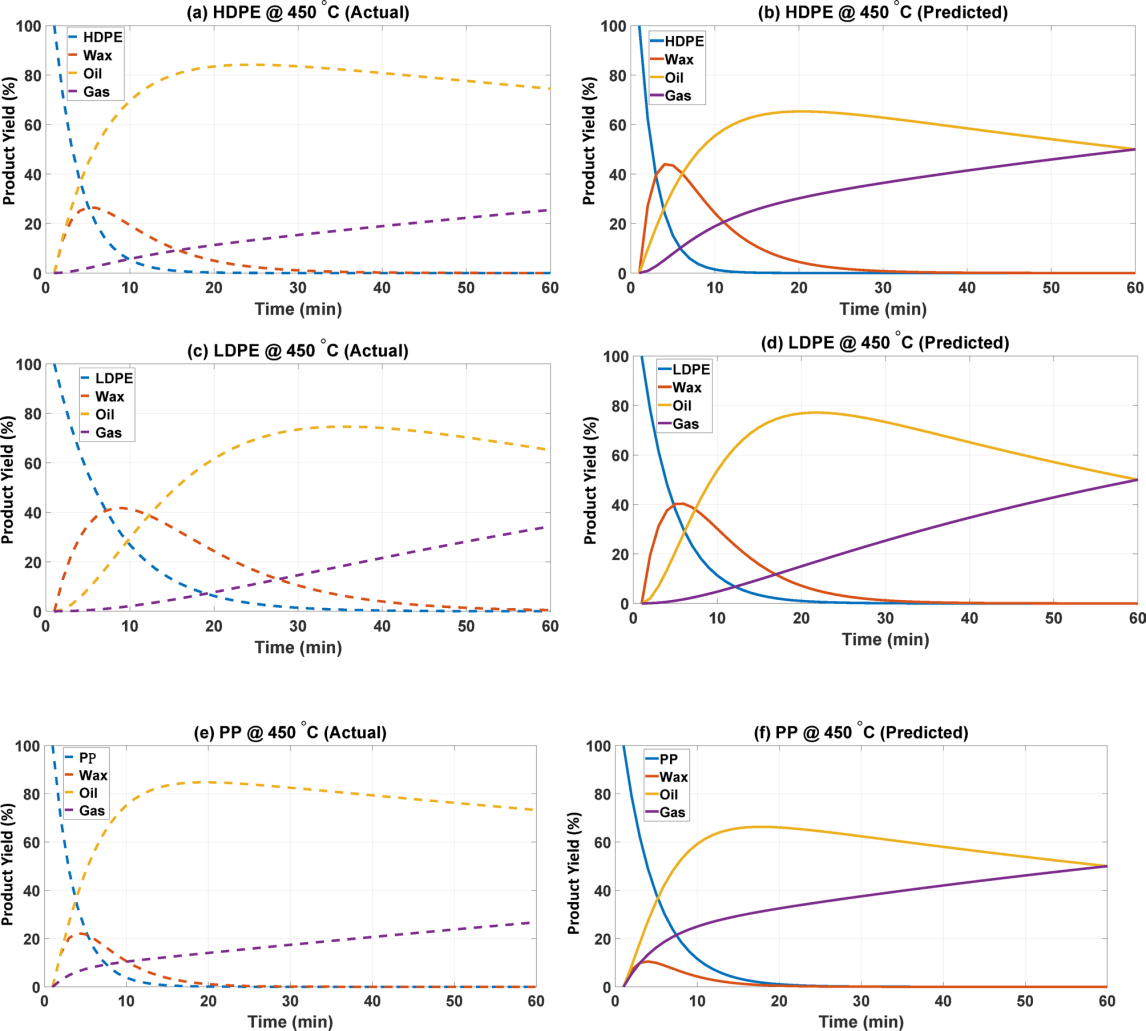
Actual versus predicted yield profiles at 450 °C using estimated kinetic rate constants.

**Fig. 4:**
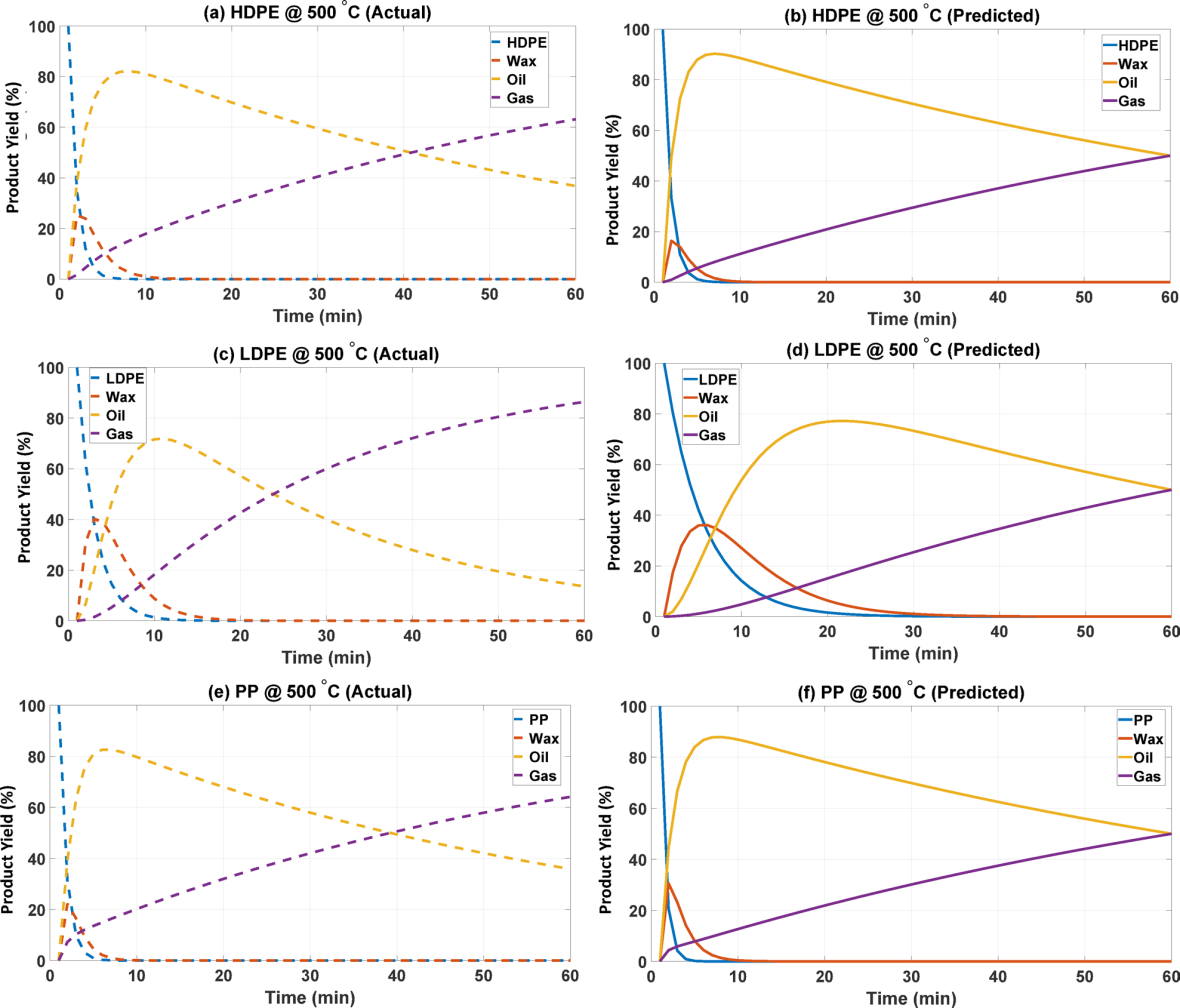
Actual versus predicted yield profiles at 500 °C using estimated kinetic rate constants.

The comparison of the actual and predicted yield distributions of HDPE, LDPE, and PP at 450 and 500 °C is illustrated in Figs. 3 and 4 to analyze the temporal evolution of yields for all three products. The yields of wax, polymer, oil, and gas products, along with all transient and steady states during polymer (oil) consumption and wax (gas) synthesis, signify the anticipated synthetic pathway for the conversion of polymer to oil and gas^15^. The model was verified at two selected temperatures, 450 and 500 °C, spanning the active pyrolysis range of polyolefins. These temperatures were chosen to investigate the decomposition behavior under both moderate and high-temperature conditions. Although simulations at other temperatures were not explicitly performed, the inverse numerical framework can be easily extended to different temperature conditions by re-optimizing the kinetic parameters using the same objective function and solver settings.

At 450 °C (refer to Fig. 3), decomposition proceeds at a comparatively slower rate, allowing for the observation of intermediates^16^. In the case of HDPE, the polymer fraction decreases swiftly during the first 10 min, indicating an immediate primary chain scission as illustrated in Fig. 3(a). Wax reaches a slight peak (about 20%) before declining, indicating a transitory intermediate produced by mid-chain cleavage^17^. Oil constitutes the primary product, peaking at around 80% within 15 min, subsequently experiencing a gradual decline due to secondary conversion processes. Gas is accumulated at a diminished rate, approximately 20% after one hour. Figure 3 (b) illustrates the anticipated profile of this transformation history; nevertheless, oil dominates (about 65% compared to 80%), wax has been reduced earlier than experimentally recorded, and gas is overrepresented (approximately 35% versus 20%). The discrepancies indicate that the model overestimates the wax-to-gas conversion via the k_6_ pathway and the oil-to-gas conversion via the k_5_ pathway. Several factors undoubtedly influence the process, including temperature gradients and regulations on heat supply within the reactor, which may prevent secondary cracking in procedure^18–20^. Vapor pressure and phase-related issues will prolong the condensation of waxes and heavy oligomers, in opposition to the assumptions regarding the formation of gases. Radical recombination and reactive reserves pathways likely overlooked or undervalued in less expensive methodologies could enhance oil and wax yields, even though introducing lighter components^21–23^. Experimental biases, such as losses during sampling, condensation from the sampling point to the gas chromatograph inlet, or calibration errors in the gas chromatograph, can systematically underestimate oil quantities while overestimating light gases^24^.

According to Fig. 3c and d, LDPE decomposes rapidly within the initial 10–15 min, demonstrating that the model well captures the swift initiation of polymer chain scission. Similar to both plots, the wax first-present (highest peak) occurs at approximately 40% Penetration within a duration of around 10 min. In actuality, once wax is present, its lifetime is somewhat prolonged until it begins to diminish gradually, whereas in projected outcomes, it vanishes considerably more swiftly^25^. The model overestimates the rate of subsequent wax cracking, as experiments indicate it remains rather constant with heat history. The oil production exhibits a notable disparity in both cases. During the experiment, the oil level progressively rises, with the predominant component (i.e., yield) stabilizing at approximately 70% during the mid to late stages^26^. The oil peak, approximately 75%, is attained earlier in the prediction, but thereafter declines due to increased gas generation. In this scenario, gas accumulates gradually, reaching around 30% after 60 min, indicating that secondary cracking occurs at a slow rate. In contrast, the model forecasts a swift increase in gas production over time, surpassing 50% at this juncture, so corroborating the tendency of the parameterized kinetics to overestimate the generation of the light portion^27^. The comparison indicates that while the general conversion pathway (LDPE → waxes → oils → gases) is predominantly preserved in replication, the anticipated model simplifies LDPE kinetics by assuming intermediates react at an accelerated rate^28^. Similarly, in the case of PP (Fig. 3e and f) demonsrate the higher output of gas with the estimated rate constants compared to the experimentally obtained. The estimated rate constants decrease wax formation at the initial scale, leading to the further decomposition of PP to oil and later to the gaseous products.

For HDPE (Fig. 4a vs. 4b), both actual and predictive analyses indicate significant chain scission occurring during the initial minutes, suggesting considerable temperature-induced chain scission^29^. In all cases, oil is the predominant output, exceeding 80% in the short term. Nonetheless, the experimental profile indicates that oil degrades, and gas accumulates gradually over a duration of 10 min, ultimately stabilizing at over 60% by the conclusion of the trial. The model overestimates oil stability, always maintaining it above 50%, but gas growth is more gradual, only achieving around 50% at 60 min. This suggests that the model underestimates the extent of secondary cracking that converts oil to gas at elevated temperatures inside this time, resulting in a less significant alteration in products than is thermally seen. The wax fractions are inconsistent; studies show negligible wax after approximately 10 min, whilst the current prediction indicates a minor transient peak in wax before its complete removal^30^.

The trends aligned with the general reaction processes the decomposition of LDPE to wax, wax to oil, and oil to gas even though with significant quantitative disparities as illustrated in Fig. 4(c) and 4(d). Experimentally, wax is predominantly generated in the initial 5–10 min before diminishing as oil predominates. Gas development is vigorous, exceeding 80% at 60 min, while oil has diminished to below 20%, signifying that intermediates experience significant secondary cracking. In the anticipated outcomes, wax is consumed more rapidly, whereas oil exhibits a significantly higher and more stable trajectory, approaching 80% before decelerating its decline to a lower level^31^. The gas levels persist in increasing but only reach approximately 40–50% at 60 min, hence diminishing the variance from experimental results.

Figures 3 and 4 indicate that the actual and prediction pyrolysis behaviors of HDPE and LDPE at 450 °C and 500 °C exhibit notable similarities, accompanied by systematic differences. The degradation pathway is accurately forecasted for all polymers: the parent polymer is the most rapidly decomposing element, succeeded by wax as an intermediary, and oil as the principal liquid result, with gas generation escalating correspondingly over time^32^. The anticipated profiles align with experimental results about the sequence of transformations; nevertheless, they diverge in the duration and half-life of intermediates. In the experimental context, wax persists more while oil experiences a significant increase, whereas the model facilitates accelerated secondary cracking consistently, and increase gas production. The comparison of actual and predicted percentage yield as a function of temperature has been documented in Table 4.

**Table 4:** The actual and predicted percentage yields comparison as a function of temperature and polymer type.

Polymer	Temperature	Product	Actual Yield (Approx.)	Predicted Yield (Approx.)
HDPE	450 °C	Polymer (P)	0%	0%
		Wax (W)	0%	0% (decays faster)
		Oil (L)	80%	65%
		Gas (G)	20%	35%
LDPE		Polymer (P)	0%	0%
		Wax (W)	5–10%	0%
		Oil (L)	70%	60%
		Gas (G)	20%	30%
PP		Polymer (P)	0%	0%
		Wax (W)	0%	0%
		Oil (L)	80%	65%
		Gas (G)	20%	35%
HDPE	500 °C	Polymer (P)	0%	0%
		Wax (W)	0%	0%
		Oil (L)	35–40%	30–35%
		Gas (G)	60%	65–70%
LDPE		Polymer (P)	0%	0%
		Wax (W)	0%	0%
		Oil (L)	20%	20%
		Gas (G)	75%	80%
PP		Polymer (P)	0%	0%
		Wax (W)	0%	0%
		Oil (L)	35–40%	35%
		Gas (G)	60%	65–70%

The results indicate significant gasification (particularly for LDPE) and an absence of gas evolution in the predictions, alongside an overestimation of oil stability and an underestimation of gas production. The inconsistencies arise from model simplification, including the modification of rate constants from one-dimensional systems, the omission of a repulsive component in the potential that accounts for steric hindrance, and the absence of recombination and diffusion terms for radicals that stabilize intermediates in actual systems. The model accurately reflects the overall trend of the transformation route; nevertheless, it overestimates the severity of side chain cracking, highlighting the necessity of integrating polymer structure with temperature-dependent radical processes for improved predictions.

### Discussion and mechanistic interpretation of polyolefin decomposition

For PP, both polymer and wax levels remain minimal, whereas oil peaks at around 80% before abruptly declining as supercritical gas reaches 60% at the conclusion of the simulation, as illustrated in Fig. 4(e). Figure 4 (f) indicates that the projected values are comparable, although the oil-to-gas conversion rate has accelerated^33^. This contradiction underscores the importance of tertiary carbons in polypropylene, which empirically appear to stabilize liquids to a higher degree than expected by the model. Multiple mechanistic and experimental factors likely play a role: the increased stability of radicals at tertiary carbons may promote chain transfer or recombination instead of immediate heterolysis, resulting in a preference for liquid-phase products. Additionally, steric effects in these areas could prevent β-scission, leading to a gas yield that is lower than anticipated due to overly simplistic model assumptions^34^. At high temperatures, polypropylene may engage in cross-linking, cyclization, or aromatization within the condensed phase, therefore sequestering a fraction of the carbon in residues heavier than those depicted in the lumped scheme, which skews the apparent mass balance towards liquids. The impacts of phase behavior such as elevated melt viscosity, bubble entrapment, and diminished volatilization kinetics for bigger oligomers may complicate experimental gas release in contrast to the homogeneous kinetics addressed below. Furthermore, the documented gas fraction may be diminished due to experimental measurement inaccuracies (e.g., insufficient gas collection) or the condensation of lighter hydrocarbons^35^. Besides the yield-based interpretation, the predicted kinetic rate constants themselves provide valuable insight into the decomposition pathways. The inverse-modeled values indicate that the primary scission constants (k₁–k₂) increase with temperature more sharply than the literature-reported values, suggesting a stronger tendency for polymer-chain rupture when the model is constrained by target yields. The secondary cracking constants (k₄–k₆) are also noticeably higher particularly for HDPE and LDPE which explains the accelerated conversion of wax and oil into gas observed in the simulated profiles. In contrast, PP shows relatively modest increases in k₂ and k₄, consistent with the stabilizing influence of tertiary carbon radicals and its experimentally observed propensity to retain more liquid-phase products.

The predicted rate constants suggest that pyrolysis is more effective and produces higher gas yields at all temperatures compared to those derived using literature-based experimental kinetic rate constants. The inverse methodology predicated on target yield forecasting is streamlined by optimal assumptions regarding heat and mass transfer. The literature addressing the reaction mechanism lacks sufficient clarity to explain the absence of kinetic constants, particularly for reactions involving wax with gas and oil, followed by gas removal, as gaseous products typically predominate in the pyrolysis process. This methodology is significant as it illustrates that inverse kinetic modeling not only replicates the overall trajectory of polymer → wax → oil → gas but also provides insights into variations in product stability relative to experimental trends. The oil remains the dominant product over an extended time frame, while the gas exhibits a linear increase, signifying a balance between liquid retention and gas expansion. This bistable behavior signifies the model’s predictive capability and establishes a theoretical basis for optimizing processes aimed at selective oil and gas production. This methodology provides a customizable numerical framework for the distribution control of products in multistep pyrolysis reaction networks while simultaneously predicting kinetic rate constants. By altering the conversion pathways, more selectively preserve primary yields (such as oils and gases), resulting in an overall enhancement in gas yield by 5% through the reduction of wax production can be obtained. Significantly, the augmented oil output (> 50%) does not influence this adjustment method, underscoring its capability to optimize secondary pathways without altering the principal liquid product percentage.

To enhance model accuracy in depicting kinetics with complex reaction processes, temperature-dependent branching ratios for competitive β-scission and hydrogen transfer at higher temperatures should be incorporated. Specifically, radical stability and transport constraints influencing volatilization, bubble dynamics, and condensed phase reactivity must also be taken into account. These characteristics would enable the model to more accurately represent the structural diversity found in actual polymers and the operational complexity inherent in experimental systems. Nonetheless, the framework serves as a reliable technique by accurately outlining predominant pathways of polymer conversion, identifying conditions favorable to the stabilization of liquid products, and capturing the overarching pattern of heightened gas production with increased degree. This approach offers an analytical foundation for interpreting experimental results and practical suggestions for process designers, such as optimizing liquid oil yields at moderate temperatures or deliberately favoring gas-rich products under more extreme thermal conditions.

## Conclusion

This study represents the first ever report on the application of inverse kinetic modeling to the pyrolysis process of polyolefins, offering a dependable and predictive approach for optimizing product yields. Employing accurate estimations for rate constants, it delineates the comprehensive process from polymer to wax to oil to gas, along with discrepancies in product stability compared to experimental observations. This method of operation enables the selective regulation of primary products, yielding 5% more gas at the cost of minimizing wax production, without compromising oil output. These findings provide insight into conditions that stabilize liquid products or enhance gas yields. Enhanced models, incorporating temperature-dependent branching ratios, potential radical stability effects, and mass transport limitations, are anticipated to improve predictive accuracy. Nevertheless, for practical applications, the framework effectively identifies dominant conversion pathways and serves as a valuable foundation for integrated process design. The reverse modeling approach is also industrially relevant, as the framework provides a predictive tool for optimizing process parameters in plastic pyrolysis operations. By correlating yield targets with kinetic rate constants, the model can assist in adjusting reactor temperature profiles and residence times to achieve selective production of oil or gas. These optimization features are valuable for scaling batch or continuous reactors toward stable, high-yield operation with minimal byproduct formation. In conclusion, this strategy offers a novel and adjustable way to direct experiments and target products in complicated polyolefin pyrolysis, which is considered unique.

## Data Availability

All data generated or analyzed during this study are included in this published article.
